# Bidirectional Crosstalk between C5a Receptors and the NLRP3 Inflammasome in Macrophages and Monocytes

**DOI:** 10.1155/2016/1340156

**Published:** 2016-06-07

**Authors:** Mikel D. Haggadone, Jamison J. Grailer, Fatemeh Fattahi, Firas S. Zetoune, Peter A. Ward

**Affiliations:** Department of Pathology, University of Michigan Medical School, Ann Arbor, MI 48109, USA

## Abstract

C5a is an inflammatory mediator generated by complement activation that positively regulates various arms of immune defense, including Toll-like receptor 4 (TLR4) signaling. The NOD-like receptor pyrin domain-containing protein 3 (NLRP3) inflammasome is activated by pathogen products and cellular/tissue damage products and is a major contributor of IL-1*β*. In this study, we investigate whether C5a modulates lipopolysaccharide- (LPS-) induced NLRP3 inflammasome activation in myeloid cells. Appearance of plasma IL-1*β* during endotoxemia was reduced in C5aR1^−/−^ mice when compared to wild-type mice. In vitro, C5a significantly enhanced LPS-induced production of IL-1*β* in bone marrow Ly6C-high inflammatory monocytes, accompanied by augmented intracellular pro-IL-1*β* expression. This effect was abolished during p38 blockade by SB 203580 and in the absence of C5aR1. Conversely, C5a suppressed LPS-induced macrophage production of IL-1*β*, which was accompanied by attenuated levels of pro-IL-1*β*, NLRP3, and caspase-1 expression. C5a's suppressive effects were negated during phosphoinositide 3-kinase (PI3K) inhibition by wortmannin but were largely preserved in the absence of C5aR1. Thus, C5a bidirectionally amplifies TLR4-mediated NLRP3 inflammasome activation in monocytes while suppressing this pathway in macrophages. However, as C5aR1 deficiency attenuates the IL-1*β* response to LPS challenge in vivo, our results suggest overall that C5a augments physiologic inflammasome responses.

## 1. Introduction

Myeloid-derived, innate inflammatory cells provide early defense against invading pathogens by activating a diverse array of protective immune mechanisms. Underlying these cells' effector functions are the pattern recognition receptors (PRRs), which detect repeating molecular motifs inherent to various pathogens (pathogen-associated molecular patterns (PAMPs)) and danger-associated molecular patterns (DAMPs) generated as a result of tissue and cellular damage. A prominent PRR known to elicit inflammation in response to gram-negative bacterial stimuli is the Toll-like receptor 4 (TLR4). Recognition of lipopolysaccharide (LPS) by this receptor activates inflammatory signaling pathways to induce the expression of numerous antimicrobial and proinflammatory molecules. An important downstream target of TLR4 is the NOD-like receptor pyrin domain-containing protein 3 (NLRP3) inflammasome [1]. This multiprotein complex, responsible for cell pyroptosis and the caspase-1-dependent processing of pro-IL-1*β* and pro-IL-18 to their biologically active forms [2], has been shown to play a prominent role in regulating both chronic and acute inflammation as a responder to cell damage and stress. Induction of the NLRP3 inflammasome is understood to proceed via a two-step process. Signal 1 [LPS and tumor necrosis factor (TNF)-*α*, etc.] serves as a priming stimulus by transcriptionally activating NLRP3 and pro-IL-1*β* expression, thus inducing the production of essential inflammasome components [1]. Recent work, however, has also suggested this priming step to involve nontranscriptional regulation [3, 4]. Signal 2 then activates the inflammasome by inducing cationic fluxes (K^+^ efflux and elevated intracellular Ca^+2^), mitochondrial and lysosomal damage, and the subsequent generation of cathepsins and reactive oxygen species (ROS) to elicit its effector functions [1, 2]. This activation step can be triggered by a large number of both endogenous [adenosine triphosphate (ATP), alum, monosodium urate, and histones, etc.] and exogenous (pore-forming toxins and bacterial RNA, etc.) stimuli [1, 2]. Thus, it is the integration of multiple signals that activates the NLRP3 inflammasome to provide for a robust inflammatory response.

The complement anaphylatoxin C5a is known to be a potent regulator of acute inflammatory responses and has been implicated to play a role in manifesting numerous inflammatory diseases, such as sepsis [5, 6]. C5a has two known receptors, C5aR1 (CD88) and C5aR2 (GPR77), which signal through phosphoinositide 3-kinase (PI3K), p38, and nuclear factor (NF)-*κ*B, amongst other pathways [5]. It is well understood that C5a binds to and signals through C5aR1, a cell-surface G protein-coupled rhodopsin-like receptor [7], though the role of C5aR2 remains controversial [8–10]. However, even with C5aR2's enigmatic function, C5a interactions with both receptors are important for eliciting the pathophysiology observed during sepsis [11].

Despite extensive efforts made to describe the crosstalk between C5a and TLR4 signaling [12–15], no work to date has illuminated the ability of C5a to regulate TLR4-mediated NLRP3 inflammasome activation. Furthermore, studies illustrating the interplay between complement and the inflammasome remain limited [16–18]. In this study, we describe a mechanism by which C5a differentially modulates TLR4-induced NLRP3 inflammasome function in macrophages and monocytes. Notably, C5a suppressed LPS-induced NLRP3, IL-1*β*, and caspase-1 expression in mouse macrophages, leading to attenuated IL-1*β* secretion following NLRP3 activation. This effect appeared to be reliant upon PI3K signaling. Conversely, acting through the mitogen-activated protein (MAP) kinase p38, C5a augmented the production of IL-1*β* in LPS-stimulated, Ly6C^+^ monocytes. While this latter effect appeared to be entirely dependent upon C5aR1 signaling, our data surprisingly suggested that C5a's suppression of the NLRP3 inflammasome in mouse macrophages might occur with limited dependency on C5aR1 engagement. Together, these findings provide significant insight into the immunomodulatory role of C5a during acute inflammation in vivo, as wild-type mice produced significantly more IL-1*β* during sublethal endotoxemia than their C5aR1^−/−^ counterparts.

## 2. Materials and Methods

### 2.1. Animals

Procedures performed in this study were all in accordance with the US National Institutes of Health guidelines and were approved by the University of Michigan Committee on the Use and Care of Animals. Experiments were performed in male, age-matched C57BL/6 mice (10–12 weeks old) purchased from Jackson Laboratories (Bar Harbor, ME, USA) and C5aR1^−/−^ mice bred and genotyped in facilities at the University of Michigan (Ann Arbor, MI, USA). All mice were housed in pathogen-free conditions with free access to food and water.

### 2.2. Reagents

Recombinant mouse C5a (R&D Systems, Minneapolis, MN, USA), LPS (*Escherichia coli*, o111:B4), adenosine triphosphate (ATP, both from Sigma-Aldrich, St. Louis, MO, USA), wortmannin (Santa Cruz Biotechnology, Santa Cruz, CA, USA), and SB 203580 (InvivoGen, San Diego, CA, USA) were all used at concentrations indicated in the figures.

### 2.3. Sublethal Endotoxemia

Mice received a 10 mg/kg body weight intraperitoneal (i.p.) injection of LPS. Plasma was harvested after 8 hours by bleeding from the retroorbital venous plexus under isoflurane anesthesia.

### 2.4. In Vitro Assays

Mouse peritoneal macrophages were harvested via the instillation and retraction of 8 mL sterile PBS (Life Technologies, Carlsbad, CA, USA) 4 days following i.p. injection of 1.5 mL 2.4% thioglycollate (Life Technologies). Mouse peritoneal neutrophils were isolated 4 hours after an i.p. administration of thioglycollate via peritoneal lavage, as described above. Total bone marrow cells were harvested by flushing bilateral femurs with PBS, and erythrocytes were lysed in hypotonic buffer. All cells were cultured in RPMI 1640 media (Life Technologies) supplemented with 100 U/mL penicillin-streptomycin and 0.1% BSA. Cell-free supernatants were stored at −80°C until later use.

To achieve NLRP3 inflammasome activation and IL-1*β* secretion in vitro, 1 × 10^6^ cells were incubated with LPS in the copresence or absence of C5a for 4 hours followed by stimulation with ATP for 45 minutes. In select experiments, cells were pretreated with signaling inhibitors 1 hour prior to the addition of LPS and/or C5a. For mRNA expression studies, total RNA was harvested from cultured cells after LPS treatment with or without C5a for 4 hours.

### 2.5. Enzyme-Linked Immunosorbent Assays (ELISA)

Mouse IL-1*β* was detected using sandwich ELISA (R&D Systems). All protocols were performed according to the manufacturer's recommendations. Cytokine concentrations were determined from log-transformed standard concentrations plotted on a standard curve.

### 2.6. Western Blotting

Estimations of supernatant protein levels were determined by bicinchoninic acid assay (Thermo Fisher Scientific, Waltham, MA, USA), and 50 *μ*g total protein was loaded per well for PAGE. Supernatant protein was separated by SDS-PAGE and transferred onto nitrocellulose membranes (Bio-Rad, Hercules, CA, USA). IL-1*β* was detected using anti-mouse IL-1*β* antibodies (R&D Systems) followed by peroxidase-conjugated, rabbit anti-goat IgG antibodies (Jackson ImmunoResearch Laboratories, West Grove, PA, USA). Detection of IL-1*β* protein was visualized with chemiluminescent substrate (Thermo Fisher Scientific).

### 2.7. Quantitative Real-Time Polymerase Chain Reaction (qRT-PCR)

Total RNA was isolated from cultured macrophages and bone marrow cells using TRIzol (Sigma-Aldrich). Following purification, DNAse (Life Technologies) was used to remove any contaminating genomic DNA. cDNA was generated using the reverse transcription kit provided by Life Technologies, and RT-PCR (SYBR, Life Technologies) was performed on a 7500 real-time PCR system (Applied Biosystems, Foster City, CA, USA). The following primers were used: GAPDH, 5′-CTTCAACAGCAACTCCCACTCTTCC-3′ (forward), and 5′-GGTGGTCCAGGGTTTCTTACTCC-3′ (reverse); NLRP3, 5′-GAGTTCTTCGCTGCTATGT-3′ (forward) and 5′-ACCTTCACGTCTCGGTTC-3′ (reverse); IL-1*β*, 5′-CCTGCTGGTGTGTGACGTTC-3′ (forward) and 5′-CAGCACGAGGCTTTTTTGTTGT-3′ (reverse); ASC, 5′-GAAGCTGCTGACAGTGCAAC-3′ (forward), and 5′-GCCACAGCTCCAGACTCTTC-3′ (reverse); Caspase-1, 5′-AGATGGCACATTTCCAGGAC-3′ (forward), and 5′-GATCCTCCAGCAGCAACTTC-3′ (reverse). Relative expression levels, normalized to GAPDH, were calculated using the 2^−ΔΔCt^ method.

### 2.8. Flow Cytometry

Cells were analyzed on a BD LSR-II flow cytometer equipped with FACSDiva software (both from BD Biosciences, San Jose, CA, USA). FlowJo 7.6.4 software (Tree Star, Ashland, OR, USA) was used for data analysis. The following antibodies were used for cell-surface labeling: PE-anti-mouse CD45, PE-Cy7-anti-mouse CD11b (both from eBioscience, San Diego, CA, USA), APC-Cy7-anti-mouse Ly6C, and PerCP-Cy5.5-anti-mouse Ly6G (both from BD Biosciences). APC-anti-mouse IL-1*β*/IL-F12 (R&D Systems) was used for the intracellular labeling of IL-1*β* in fixed and permeabilized (saponin) cells.

### 2.9. Statistical Analysis

Data in this study (values expressed as means ± standard error of the mean) were analyzed using GraphPad Prism 6 graphing and statistical analysis software (GraphPad Inc., La Jolla, CA, USA). Significance between multiple sample means was determined by one-way ANOVA followed by Tukey's multiple comparisons test. Where appropriate, significance between individual sample means was determined by two-tailed Student's *t*-test. Differences were considered significant with a* p* value < 0.05.

## 3. Results

### 3.1. C5aR1 Deficiency Attenuates IL-1*β* Buildup during Sublethal Endotoxemia

**Figure 1:**
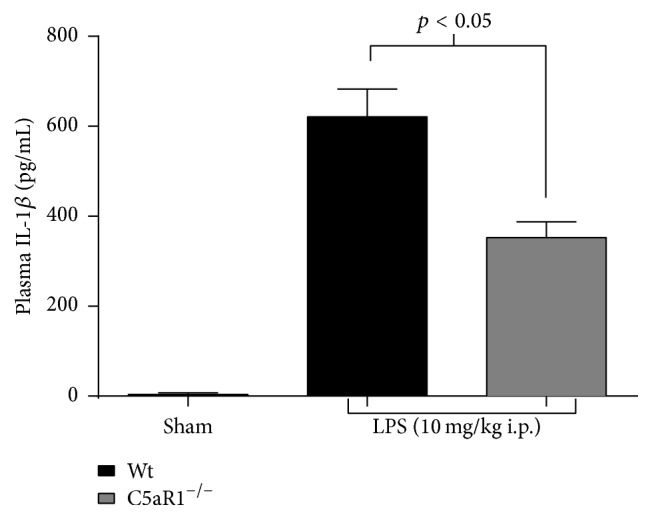
C5aR1 augments the IL-1*β* response during LPS-induced endotoxemia. Wild-type and C5aR1^−/−^ mice (*n* ≥ 5 mice per group) were injected i.p. with LPS (10 mg/kg body weight) and plasma was harvested after 8 hours. IL-1*β* production was determined by ELISA with plasma protein levels expressed as means ± standard error of the mean. Results showed that a loss of C5aR1 in vivo attenuates the LPS-activated IL-1*β* response. Wt = wild type.

Given the importance of C5a-C5aR1 interactions in promoting the onset and development of sepsis [19], we sought to investigate the regulation of IL-1*β* production by C5aR1 signaling in a TLR4-mediated, mouse endotoxemia model. For this model, wild-type and C5aR1^−/−^ mice received an i.p. injection of LPS (10 mg/kg body weight), and plasma was harvested 8 hours later. As shown in Figure 1, LPS challenge in vivo strongly induced IL-1*β* production in wild-type mice, which was significantly attenuated in the absence of C5aR1. Thus, this 43% reduction in plasma IL-1*β* detected during LPS-induced endotoxemia—attributable to a loss of C5aR1 signaling—strongly suggested that C5a engages C5aR1 in vivo to amplify TLR4-mediated IL-1*β* production in the endotoxemia model.

### 3.2. C5a Bidirectionally Regulates NLRP3 Inflammasome Activation in Macrophages and Monocytes

**Figure 2:**
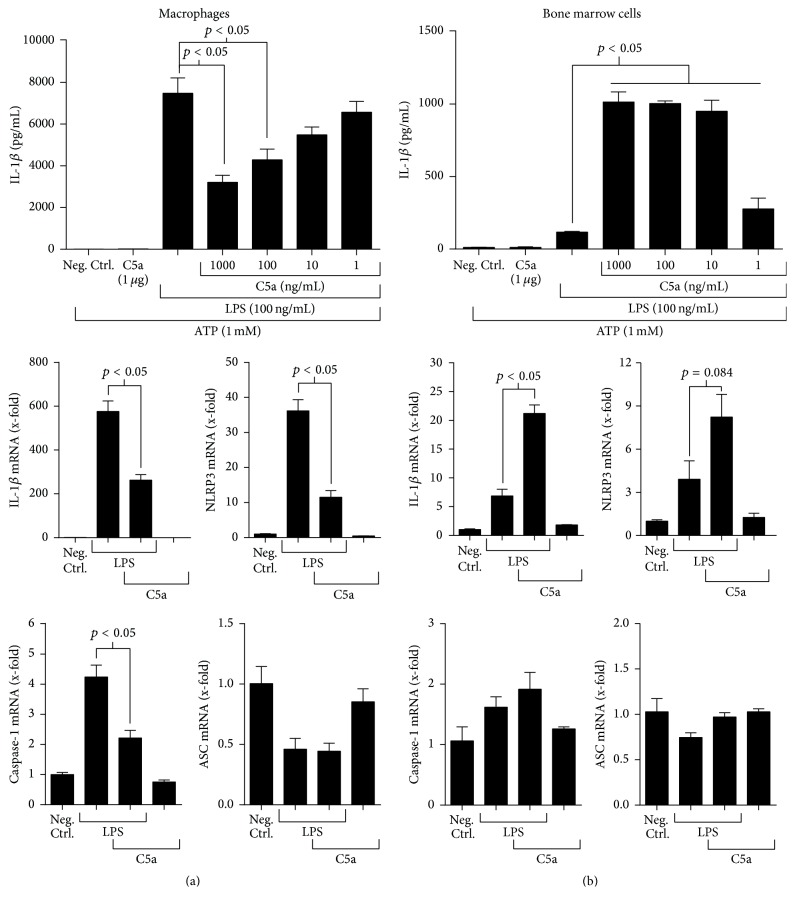
C5a suppresses NLRP3 function in macrophages but augments LPS-primed IL-1*β* production in bone marrow cells. For quantifying IL-1*β* release, 1 × 10^6^ thioglycollate-elicited peritoneal macrophages (a) or total bone marrow cells (b) were harvested, treated with LPS (100 ng/mL) in the absence or copresence of varying C5a concentrations for 4 hours, and then stimulated with ATP (1 mM) for 45 minutes to achieve NLRP3 inflammasome activation. IL-1*β* levels in cell-free supernatants were determined using ELISA (top panels). qRT-PCR assays (middle and bottom panels) were performed on mRNA isolated from 1 × 10^6^ peritoneal macrophages (a) or bone marrow cells (b) 4 hours after culture with or without LPS (100 ng/mL) in the presence or absence of C5a (1,000 ng/mL). ATP treatment was not performed for mRNA quantification experiments. Relative expression levels of inflammasome component genes (indicated as fold change) were normalized to GAPDH expression and calculated using the 2^−ΔΔCt^ method. ELISA and qRT-PCR data are shown as mean values ± standard error of the mean. Experiments were performed in triplicate for ≥2 independent experiments, and representative data are shown. Results indicated that C5a attenuates TLR4-mediated macrophage IL-1*β* production but enhances NLRP3 function in bone marrow cells. Neg. Ctrl. = negative (unstimulated) control.

Assembly of the NLRP3 inflammasome is a major prerequisite to IL-1*β* secretion in innate inflammatory cells that have been activated [20]. Given our observation that C5a modulated LPS-induced IL-1*β* production in vivo (Figure 1), we sought to determine whether C5a functionally regulates NLRP3 inflammasome activation in macrophages. We harvested thioglycollate-elicited peritoneal macrophages and treated these cells with LPS in the absence and copresence of various concentrations of C5a for 4 hours. Using the inflammasome protocol, macrophages were then stimulated with ATP for 45 minutes to achieve NLRP3 activation. IL-1*β* secretion was subsequently quantified by ELISA in supernatant fluids of macrophages. In a dose-dependent manner, C5a significantly suppressed LPS-induced macrophage IL-1*β* release (Figure 2(a) (top panel) and Supplementary Figure 1(a) in Supplementary Material available online at http://dx.doi.org/10.1155/2016/1340156). These data suggested that C5a suppresses TLR4-mediated inflammasome priming in macrophages, leading to attenuated IL-1*β* secretion. To explore this hypothesis, we investigated the effects of C5a on the expression of pro-IL-1*β*, NLRP3, caspase-1, and ASC (apoptosis-associated speck-like protein containing a carboxy-terminal CARD), an adapter protein that bridges the NLRs and caspase-1 [21]. For these mRNA expression studies, we withheld ATP treatment to specifically investigate C5a's effects on the intracellular expression, rather than activation, of inflammasome machinery. In agreement with our IL-1*β* protein data, the addition of C5a significantly dampened pro-IL-1*β*, NLRP3, and caspase-1 mRNA expression levels in macrophages activated by LPS (Figure 2(a), middle and bottom panels). However, changes in ASC mRNA were refractory to LPS and C5a cotreatment, and no effect was observed in the presence of C5a alone (Figure 2(a), middle and bottom panels). These findings suggested that C5a reduces TLR4-mediated macrophage IL-1*β* production via suppressed NLRP3 inflammasome priming. However, our data could not directly reconcile the observation that C5aR1 deficiency suppressed the in vivo IL-1*β* response (Figure 1).

As the inflammatory response to endotoxin challenge involves several innate inflammatory cell types (e.g., macrophages, monocytes, and neutrophils), we considered the possibility that C5a may bidirectionally regulate NLRP3 inflammasome activation throughout the myeloid cell compartment to augment LPS-induced IL-1*β* production in vivo. To explore this, we harvested bone marrow cells from wild-type mice and induced inflammasome activation via cotreatment with LPS followed by ATP in the absence and copresence of various concentrations of C5a. As was the case for our macrophage studies, C5a was administered concomitantly with LPS to investigate its potential regulation of inflammasome priming. Interestingly, C5a robustly enhanced TLR4-mediated NLRP3 function in total bone marrow cells, and the magnitude of this response was largely sustained even upon treatment with a low dose (10 ng/mL) of C5a (Figure 2(b), top panel). Detection of the processed form (17 kDa), but not unprocessed form (31 kDa), of IL-1*β* confirmed inflammasome activation under these cell culture conditions, albeit to a lesser extent than that observed for macrophages (Supplementary Figure 1). Accordingly, qRT-PCR assays performed under ATP-deficient conditions revealed that the addition of C5a significantly increased LPS-activated pro-IL-1*β* expression while also eliciting a nonsignificant, yet notable increase in NLRP3 mRNA levels under these conditions (Figure 2(b), middle panel). However, no changes in caspase-1 and ASC expression were observed, and no effect was observed in the presence of C5a alone (Figure 2(b), middle and bottom panels). Taken together, our results indicated that C5a promotes IL-1*β* production downstream of TLR4 activation in bone marrow cells, at least in part via transcriptional modulation of the NLRP3 inflammasome. As this effect was opposite the effect observed in macrophages (Figure 2(a)), our data strongly suggested that C5a can bidirectionally regulate inflammasome responses throughout the myeloid cell compartment depending on the myeloid-derived cell type.

**Figure 3:**
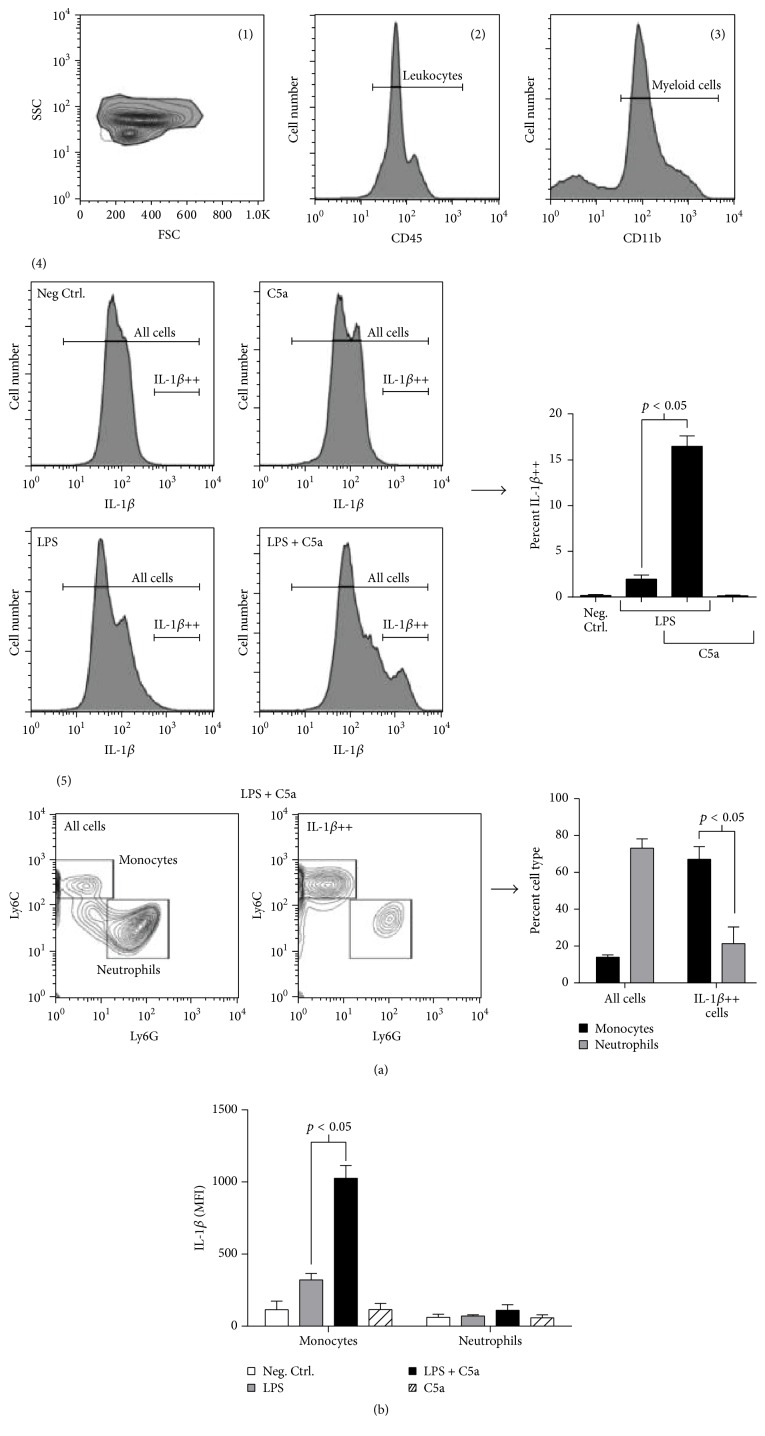
C5a robustly enhances TLR4-mediated IL-1*β* production in bone marrow inflammatory monocytes but not neutrophils. 1 × 10^6^ bone marrow cells were plated and cultured for 4 hours with or without LPS (100 ng/mL) in the absence or presence of C5a (1,000 ng/mL). Intracellular IL-1*β* staining was performed to determine the relative contributions of monocytes (CD11b^+^Ly6C^+^) and neutrophils (CD11b^+^Ly6G^+^) to the elevated bone marrow IL-1*β* response observed during LPS and C5a cotreatment (a). IL-1*β* MFI was measured to quantify the effect of C5a on LPS-induced IL-1*β* production (b). Numbers included in (a) refer to flow cytometry gates. Cell percentages and MFI values are expressed as means ± standard error of the mean. Experiments were performed in triplicate for ≥2 independent experiments, and representative results are shown. Data indicated that inflammatory monocytes in the bone marrow are uniquely sensitive to C5a's NLRP3-enhancing effects. MFI = mean fluorescence intensity; Neg. Ctrl. = negative (unstimulated) control.

Since the bone marrow contains multiple cell types within the myeloid lineage, we next wanted to delineate which bone marrow cells were responsible for mediating C5a's enhancement of NLRP3 function. Monocytes and neutrophils are derived from a common myeloid progenitor in the bone marrow, and both contribute to pathogen clearance by activating inflammatory pathways. Peripheral monocytes can be further subdivided into two major phenotypes based on the expression of Ly6C [22, 23]. After being released from the bone marrow, Ly6C^+^ inflammatory monocytes rapidly traffic to sites of inflammation where they respond to microbial stimuli and are thought to, over several days, differentiate into macrophages and dendritic cells [22, 23]. These cells are also capable of giving rise to Ly6C^low^ monocytes, which are similarly recruited to sites of inflammation, but they appear to respond with different functions [22]. Thus, we decided to determine whether Ly6C^+^ inflammatory monocytes or neutrophils in the bone marrow could demonstrate C5a-enhanced inflammasome responses. To investigate this, bone marrow cells were isolated from wild-type mice and treated with LPS in the absence and copresence of C5a for 4 hours. Cells were then harvested and analyzed by flow cytometry to determine the relative contributions of cells to IL-1*β* production by bone marrow monocytes and neutrophils. Our gating strategy is outlined in Figure 3(a): within the CD45^+^ leukocyte gate (gate 2), myeloid cells were first identified by CD11b expression (gate 3), and IL-1*β* was detected intracellularly (gate 4). When bone marrow cells were cotreated with LPS and C5a, a subset of myeloid cells demonstrated an IL-1*β*-high (IL-1*β* ^++^) phenotype, which was absent in the presence of either LPS or C5a alone (Figure 3(a)). Monocytes and neutrophils were further defined by their expression of Ly6C (CD11b^+^Ly6C^+^) and Ly6G (CD11b^+^Ly6G^+^), respectively (gate 5). By determining the cell-surface phenotype of IL-1*β* ^++^ cells, our data revealed that most IL-1*β*-containing cells were CD11b^+^Ly6C^+^, whereas neutrophils represented only a small proportion of this IL-1*β* ^++^ population despite constituting the large majority of bone marrow CD11b^+^ cells (gate 5). Furthermore, analyses of IL-1*β* mean fluorescence intensity (MFI) demonstrated that monocytes had significantly upregulated LPS-induced IL-1*β* production when cotreated with C5a, whereas TLR4-mediated, neutrophil-specific IL-1*β* production was not sensitive to C5a stimulation (Figure 3(b)). A similar result was observed when we stimulated thioglycollate-elicited peritoneal neutrophils with LPS and ATP in in copresence of C5a. Although C5a did elicit a significant increase in neutrophil IL-1*β* secretion under these conditions (Supplementary Figure 2), its effects were modest when compared to the response exhibited by monocytes. Taken together, our results suggested that Ly6C^+^ inflammatory monocytes were the major cell type in the bone marrow to augment IL-1*β* production when cotreated with LPS and C5a, whereas the contribution to this response by neutrophils was marginal. Therefore, these data suggest that Ly6C^+^ monocytes, known to be released from the bone marrow during sepsis [24], are the target of C5a in vivo, which results in elevated IL-1*β* production and release. However, our data do not exclude the possibility that C5a augments NLRP3 inflammasome function in these cells through an indirect, cell-extrinsic mechanism. As other myeloid subsets, including polymorphonuclear leukocytes (PMNs), are known to activate in response to C5a, it is possible that the inflammatory milieu elicited by C5a, rather than C5a itself, is responsible for enhanced monocyte IL-1*β* production under these conditions. Exploration into a potential C5a-induced myeloid cell crosstalk mechanism will require follow-up experimentation.

### 3.3. Divergent C5a-Induced Signaling in Macrophages and Bone Marrow Cells Results in Different Effects on IL-1*β* Production

**Figure 4:**
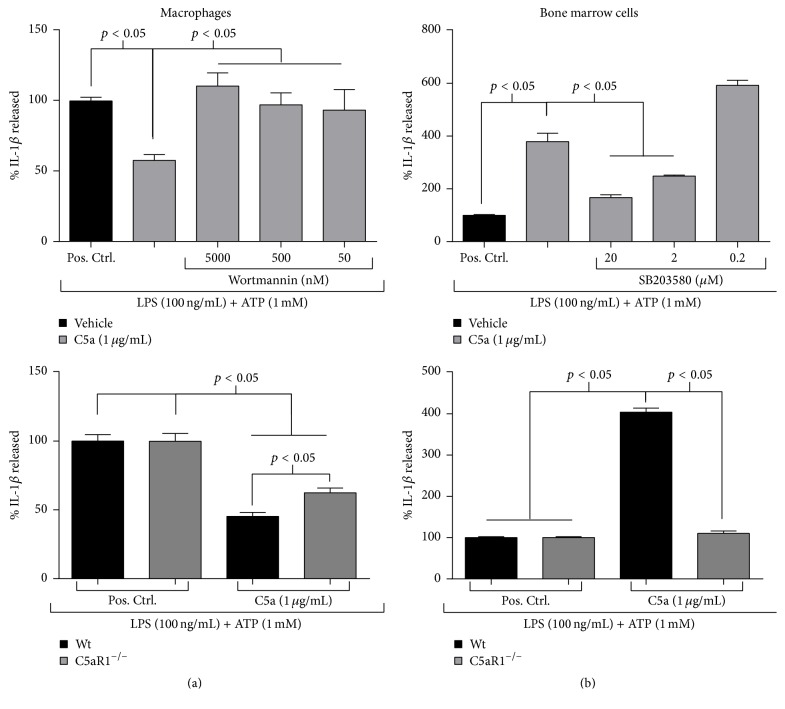
C5a augments LPS-primed IL-1*β* production in bone marrow cells via C5aR1 and p38 signaling but suppresses the NLRP3 pathway in macrophages through PI3K with limited dependency on C5aR1. For signaling inhibition studies (top panels), 1 × 10^6^ peritoneal macrophages (a) or bone marrow cells (b) harvested from wild-type mice were pretreated with the indicated concentrations of wortmannin (a) or SB 203580 (b) for 1 hour prior to LPS (100 ng/mL) stimulation (4 hours) in the absence or copresence of C5a (1,000 ng/mL). IL-1*β* secretion, as quantified by ELISA, was elicited by subsequently treating cells with ATP (1 mM) for 45 minutes. Percentage of IL-1*β* release was calculated relative to positive control samples (LPS and ATP stimulation in the absence of C5a) for each concentration of inhibitor tested. For studies investigating C5aR1 dependency (bottom panels), 1 × 10^6^ peritoneal macrophages (a) or bone marrow cells (b) harvested either from wild-type or C5aR1^−/−^ mice were treated with LPS (100 ng/mL) in the absence or copresence of C5a (1,000 ng/mL) for 4 hours. ELISA was used to quantify supernatant IL-1*β* levels following subsequent ATP (1 mM) treatment for 45 minutes. Percent IL-1*β* release was calculated relative to positive control samples (LPS and ATP treatment in the absence of C5a) for each mouse strain tested. All values are expressed as means ± standard error of the mean. Experiments were performed in triplicate for ≥2 independent experiments, and pooled data are shown. Data indicated that C5a requires p38 and C5aR1 for enhancing NLRP3 function in bone marrow cells but does not fully depend on C5aR1 for suppressing the LPS-primed macrophage IL-1*β* response, which it regulates via PI3K signaling. Wt = wild type; Pos. Ctrl. = positive control.

C5a stimulation of immune cells activates both the PI3K/Akt and MAPK/ERK (extracellular signal-regulated kinase) signaling pathways [9, 12–15]. As PI3K is known to mediate the anti-inflammatory effects of C5a on macrophage TLR4 signaling [12–14, 25], we first investigated whether this pathway was also involved in C5a's suppression of the NLRP3 inflammasome by using wortmannin, a selective PI3K inhibitor. As shown in Figure 4(a) (upper panel) and Supplementary Figure 3(a), all concentrations of wortmannin tested (5,000, 500, and 50 nM) reduced the suppressive effects of C5a on LPS- and ATP-induced macrophage IL-1*β* secretion. Thus, these data suggested that PI3K is required for C5a's inhibition of the IL-1*β* response. However, when investigating C5a's reliance on C5aR1 signaling for mediating its inflammasome-suppressing effects, we were very surprised to find that, similar to wild-type macrophages, C5aR1^−/−^ macrophages demonstrated dampened IL-1*β* production after treatment with LPS and ATP in the copresence of C5a (Figure 4(a) (lower panel) and Supplementary Figure 3(b)). Relative to positive control samples, C5a attenuated IL-1*β* secretion by 38% in C5aR1^−/−^ macrophages as compared to 55% in wild-type macrophages, thus representing only a modest loss of inhibition. These data demonstrated that C5aR1 is dispensable for C5a to suppress TLR4-driven inflammasome activation. This mechanism contrasts those elucidated in our previous studies, which have all implicated C5aR1 to be the critical C5a receptor that modulates LPS-activated macrophage cytokine responses [12–14], even when C5aR2 was shown to be functionally relevant [14].

Given this surprising result, we then wanted to explore the mechanism underlying C5a's enhancement of LPS-induced bone marrow IL-1*β* production, which we largely attributed to augmented inflammasome activation in inflammatory monocytes (Figure 3). As p38 signaling has been shown to potentiate IL-1 and TNF-*α* production in activated monocytes [26] and to mediate C5a-enhanced monocyte IL-6 secretion induced by LPS [15], we investigated whether this pathway also assumed a functional role downstream of C5a to enhance the NLRP3 inflammasome response. To investigate this, we elicited IL-1*β* secretion from bone marrow cells in the copresence of C5a and various concentrations of SB 203580 (a pharmacologic p38 inhibitor) and evaluated the effect of p38 blockade on inflammasome activation. As shown in Figure 4(b) (upper panel) and Supplementary Figure 3(c), higher concentrations of SB 203580 largely negated the increase in LPS-induced IL-1*β* production evoked by C5a costimulation. Thus, our results implicated p38 to act as a critical downstream target for C5a that elicits augmented inflammasome function in bone marrow cells. To then determine whether C5a depended on C5aR1 for potentiating the IL-1*β* response, we performed our inflammasome activation protocol on bone marrow collected from C5aR1^−/−^ mice and compared the subsequent IL-1*β* response to that observed in wild-type bone marrow cells. In contrast to our macrophage studies, C5a's enhancement of TLR4-mediated inflammasome activation in the bone marrow was entirely dependent on C5aR1 signaling (Figure 4(b) (lower panel) and Supplementary Figure 3(d)). Altogether, these data—in conjunction with our flow cytometry results (Figure 3)—strongly suggested that, for inflammatory monocytes stimulated by LPS, C5a engages C5aR1 to elicit downstream p38 signaling, which is critical for augmenting TLR4-mediated inflammasome activation and IL-1*β* production. Furthermore, as our in vivo data show (Figure 1), this C5a-enhanced inflammasome function and IL-1*β* release are likely important during experimental endotoxemia.

## 4. Discussion

In this study, we explored alterations in NLRP3 inflammasome function elicited by the complement anaphylatoxin C5a in vivo using a LPS-induced endotoxemia model and in vitro by exploring innate inflammatory cell activation of NLRP3 in the absence and presence of C5a. Interestingly, C5aR1 deficient mice had attenuated IL-1*β* expression during experimental endotoxemia, thus suggesting that C5a-C5aR1 interactions in vivo augment the LPS-activated IL-1*β* response. This is in agreement with previous studies that have described a critical role for C5aR1 engagement in exacerbating sepsis immunopathology [19]. Given this result, we hypothesized that C5a modulates TLR4 signaling throughout the myeloid compartment to enhance NLRP3 inflammasome function. Recent studies by An et al. have suggested that C5a was related to the generation of downstream monosodium urate/uric acid (MSU) crystals during sterile inflammation, resulting in activation of monocyte inflammasome priming [27]. Although macrophage regulation was not explored under these conditions, their studies showed that C5a alone stimulated human monocyte pro-IL-1*β* expression, which then required MSU crystals for maximally activating NLRP3 function. In our hands, C5a similarly enhanced IL-1*β* production in inflammatory monocytes, while suppressing this pathway in mouse macrophages through the attenuation of inflammasome transcriptional priming. However, in contrast to the mechanism proposed by An et al., we could not demonstrate that C5a by itself serves as a priming stimulus to modulate the transcription of NLRP3 machinery and/or its substrates. Rather, our observations indicated that C5a depends on the copresence of a potent priming stimulus (e.g., LPS) for regulating NLRP3 activity. A number of explanations may help to reconcile this discrepancy. One is that the mechanisms underlying C5a's regulation of the inflammasome in human and mouse monocytes might be fundamentally different. Another explanation stems from the concentration-dependency of C5a-regulated immune responses, which has been described in numerous studies. Specifically, An et al. documented pro-IL-1*β* expression occurring in human monocytes when C5a was administered at moderately low concentrations (50–100 ng/mL), whereas C5a concentrations outside of this range did not elicit significant changes in inflammasome responsiveness. In our experiments, high C5a concentrations (100–1,000 ng/mL) potently regulated LPS-activated transcriptional priming, though this C5a treatment by itself did not enhance or suppress NLRP3 function. It is worth noting that low C5a concentrations were not as effective at modulating TLR4-mediated IL-1*β* secretion as were high C5a concentrations. Taken together, we conclude that the nature of C5a-NLRP3 crosstalk is likely dependent on both the copresence/absence of canonical NLRP3 priming stimuli along with the amount of C5a generated during an inflammatory response. Specifically, An et al. suggest a “bell-curve-like” mechanism during sterile inflammation where, in the absence of PAMPs, inflammasome priming is regulated according to a small window of low C5a concentrations. However, our data show that during septic inflammation, C5a tunes strong TLR4-mediated inflammasome responses over a broader range of C5a levels with greater modulation occurring at higher concentrations. Understanding how the complex crosstalk between complement, PAMPs, and DAMPs regulates inflammation in vivo will require intense follow-up investigation.

Upon addressing the receptor dependency underlying C5a's modulatory effects, we were very intrigued to find that while C5a required C5aR1 for enhancing inflammasome-mediated monocyte IL-1*β* responses, its suppression of this pathway in macrophages was only moderately lost in the absence of C5aR1. This result suggested that C5aR2—as opposed to C5aR1—might play a more prominent role in regulating macrophage IL-1*β* release, which contrasts its function in the context of other cytokine responses [12–15]. Additional studies using C5aR2^−/−^ mice are needed to substantiate this hypothesis, though if C5aR2 is required for C5a's suppression of NLRP3 inflammasome function, we are interested to investigate the IL-1*β* phenotype of C5aR2^−/−^ endotoxemic mice. It may be that macrophage C5aR2 engagement in vivo acts to prevent LPS signaling from tipping the balance towards dysregulated inflammasome responses. As C5aR2 has been previously suggested to assume an anti-inflammatory function [28, 29], we would like to explore whether this receptor is critical for attenuating IL-1*β*-related immunopathology. However, given C5aR2's enigmatic functions, it is unclear as to why C5a might favor this receptor over C5aR1 for suppressing the inflammasome. C5aR2 has been previously shown to signal downstream to PI3K [9], which is in agreement with our signaling data, though unraveling what happens proximal to PI3K activation to allow for noncanonical receptor specificity necessitates further investigation. In addition, while our data showed that C5a differentially regulated myeloid cell NLRP3 inflammasome function by activating divergent signaling pathways—potentially due to disparate receptor dependencies—we also cannot rule out the possibility that C5aR1 expression differences amongst myeloid subsets might be important for regulating IL-1*β* release. However, as comparable levels of C5aR1 have been previously quantified on murine monocytes and thioglycollate-elicited macrophages [30], we do not expect this to be the sole causal mechanism underlying our observations.

While our in vitro studies suggested that C5a directly modulates TLR4 signaling to influence NLRP3 inflammasome transcriptional priming, it is likely that this regulation in vivo is multifaceted. Specifically, our endpoints addressed mechanisms that would largely suggest cell-intrinsic NLRP3 regulation by C5a, but we cannot rule out the possibility that C5a might modulate inflammasome activation via cell-extrinsic effects. A mechanism of interest to our group involves C5a-mediated neutrophil activation. Specifically, we have shown that the C5a generated during acute lung injury and sepsis induces a robust release of nuclear histones from neutrophils, which has both lymphodepleting [31] and pathophysiologic consequences [32, 33]. Furthermore, recent studies have characterized extracellular histones as potent activators of the NLRP3 inflammasome, behaving as both “signal 1” and “signal 2” stimuli [34–36]. Thus, the augmented IL-1*β* production elicited by C5a in our bone marrow stimulation experiments may have involved a neutrophil-driven histone release step. Similarly, it is possible that proinflammatory cytokines and chemokines released by neutrophils following C5a stimulation might also play a prominent role in regulating the downstream monocyte IL-1*β* response. These are intriguing hypotheses, as C5a-mediated inflammasome transcriptional modulation appeared to be less prominent in bone marrow cells, suggesting the potential involvement of cell-extrinsic monocyte regulatory mechanisms. Furthermore, although our data demonstrated that C5a does not potently regulate neutrophil IL-1*β* secretion, as mediators of C5a-induced histone release, these cells might play a critical role in eliciting the C5a-enhanced NLRP3 activation that we observed during physiologic endotoxemia. Studies performed under neutrophil- and/or histone-depleting conditions are required to determine the functional relevance of this pathway.

Finally, while our experiments with bone marrow cells demonstrated that NLRP3 enhancement in inflammatory monocytes was uniquely sensitive to C5a stimulation, additional in vivo work is needed to understand the contribution of these cells to the inflammatory response observed during endotoxemia. Ly6C^+^ monocytes in the bone marrow are mobilized in response to inflammatory stimuli and those coexpressing high levels of CC-chemokine receptor 2 (CCR2) traffic to sites of infection where CCR2 ligands are produced [22]. Once in tissues, these cells are capable of differentiating into a myriad of myeloid subsets with varying phenotypic and functional features, such as migratory dendritic cells (DCs), inflammatory macrophages, and/or TNF- and iNOS- (inducible nitric oxide synthase-) producing DCs [22]. Although our in vitro data suggested that inflammatory monocytes might be the target of C5a-augmented NLRP3 function during LPS challenge in vivo, it remains to be determined whether they are indeed important for robust IL-1*β* production. In addition, it would be beneficial to characterize these cells' ultimate fate, and understand whether monocyte sensitivity to C5a-enhanced inflammasome function is maintained or lost upon achieving terminal differentiation.

## 5. Conclusion

The results of our study suggest that C5a-C5aR1 interactions are critical for enhancing the innate IL-1*β* response during LPS-induced endotoxemia. In addition, our findings show for the first time that C5a bidirectionally regulates NLRP3 inflammasome activation throughout the myeloid cell compartment. Specifically, C5a engages C5aR1 to augment TLR4-mediated IL-1*β* production in inflammatory monocytes via the p38 MAPK signaling pathway, while it suppresses macrophage IL-1*β* secretion independent of C5aR1 engagement via the PI3K pathway. Follow-up experiments will be required for understanding the relative contributions of these two mechanisms in eliciting the net IL-1*β* response observed during sepsis. Taken together, our data provide novel insight into the mechanisms underlying C5a's immunomodulatory functions during acute inflammation while further characterizing NLRP3 regulation in vivo and in vitro. This crosstalk is likely to play a prominent role in regulating the IL-1*β* produced during numerous inflammatory disease states in which complement, PAMPs, and DAMPs are present.

## Supplementary Material

The supplementary materials include three figures. In Supplementary Figure 1, western blot results are presented to demonstrate detection of processed, but not unprocessed, IL-1*β* in our macrophage and bone marrow cell NLRP3 inflammasome stimulation experiments. Furthermore, these data corroborate the ELISA results presented throughout our manuscript by showing alterations in myeloid cell IL-1*β* production evoked by C5a. Supplementary Figure 2 shows that C5a only modestly regulates NLRP3 inflammasome activity in peritoneal neutrophils. Supplementary Figure 3 presents the data shown in Figure 4 as absolute amounts of IL-1*β* protein released as opposed to the percent of IL-1*β* protein released relative to positive control samples.
