# Supplementary material for: Bidirectional Crosstalk between C5a Receptors and the NLRP3 Inflammasome in Macrophages and Monocytes
Source: Mediators Inflamm. 2016 Jun 7;2016:1340156. doi: 10.1155/2016/1340156 (PMC4921141; doi:10.1155/2016/1340156)
Supplement: Supplementary file 1 — The supplementary materials include three figures. In Supplementary Figure 1, western blot results are presented to demonstrate detection of processed, but not unprocessed, IL-1β in our macrophage and bone marrow cell NLRP3 inflammasome stimulation experiments. Furthermore, these data corroborate the ELISA results presented throughout our manuscript by showing alterations in myeloid cell IL-1β production evoked by C5a. Supplementary Figure 2 shows that C5a only modestly regulates NLRP3 inflammasome activity in peritoneal neutrophils. Supplementary Figure 3 presents the data shown in Figure 4 as absolute amounts of IL-1β protein released as opposed to the percent of IL-1β protein released relative to positive control samples. [file 1340156.f1.pdf]

Supplementary Figure 1

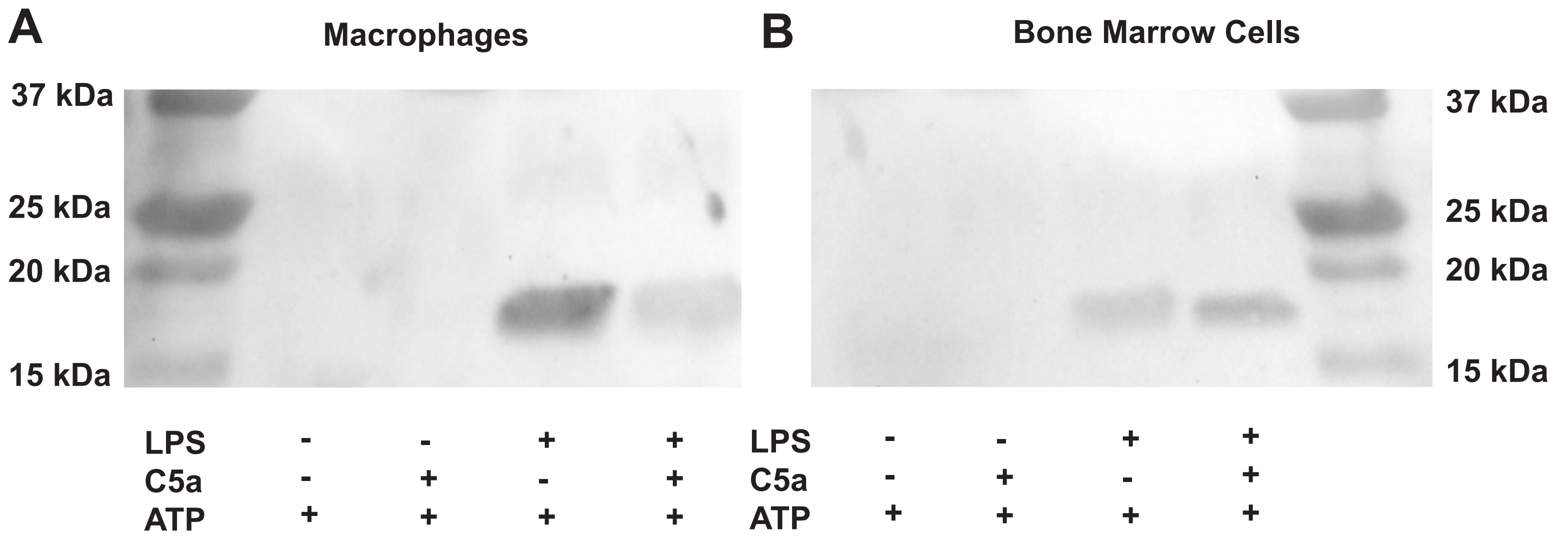

Supp. Fig. 1. Detection of processed IL-1β in macrophage and bone marrow cell culture supernatants by western blot. 1x10[6] thioglycollate-elicited peritoneal macrophages (a) or total bone marrow cells (b) were harvested, treated with LPS (100 ng/mL) in the absence or co-presence of C5a (1,000 ng/mL) for 4 hours, and then stimulated with ATP (1 mM) for 45 minutes to achieve NLRP3 inflammasome activation. Processed (17 kDa) and unprocessed (31 kDa) IL-1β levels in cell-free supernatants were determined by western blot. Results demonstrated modulation of myeloid cell IL-1β release by C5a during inflammasome stimulation.

Supplementary Figure 2

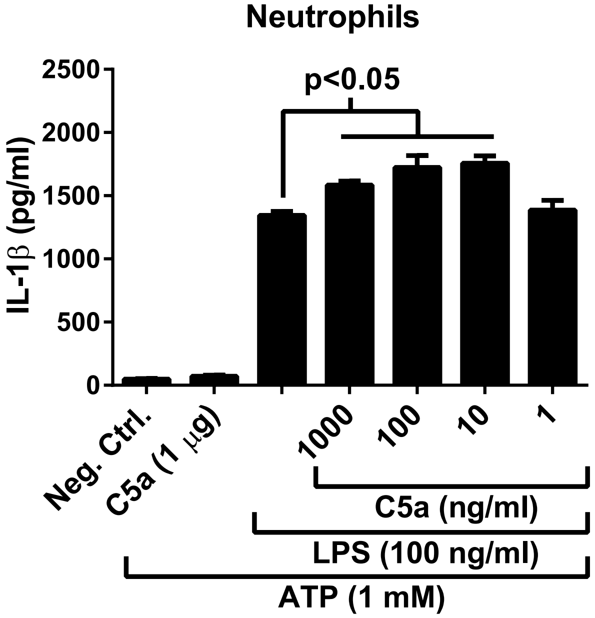

Supp. Fig. 2. C5a modestly enhances NLRP3 inflammasome function in peritoneal neutrophils. Thioglycollate-elicited peritoneal neutrophils were cultured in the presence or absence of LPS (100 ng/mL) with or without varying C5a concentrations for 4 hours, and then were stimulated with ATP (1 mM) for 45 minutes. Supernatant IL-1β levels, quantified by ELISA, are expressed as means +/- standard error of the mean. Experiments were performed in triplicate for ≥ 2 independent experiments, and representative data are shown. Data indicated that C5a only modestly enhances LPS-primed inflammasome activation in neutrophils. Neg. Ctrl. = negative (unstimulated) control.

Supplementary Figure 3

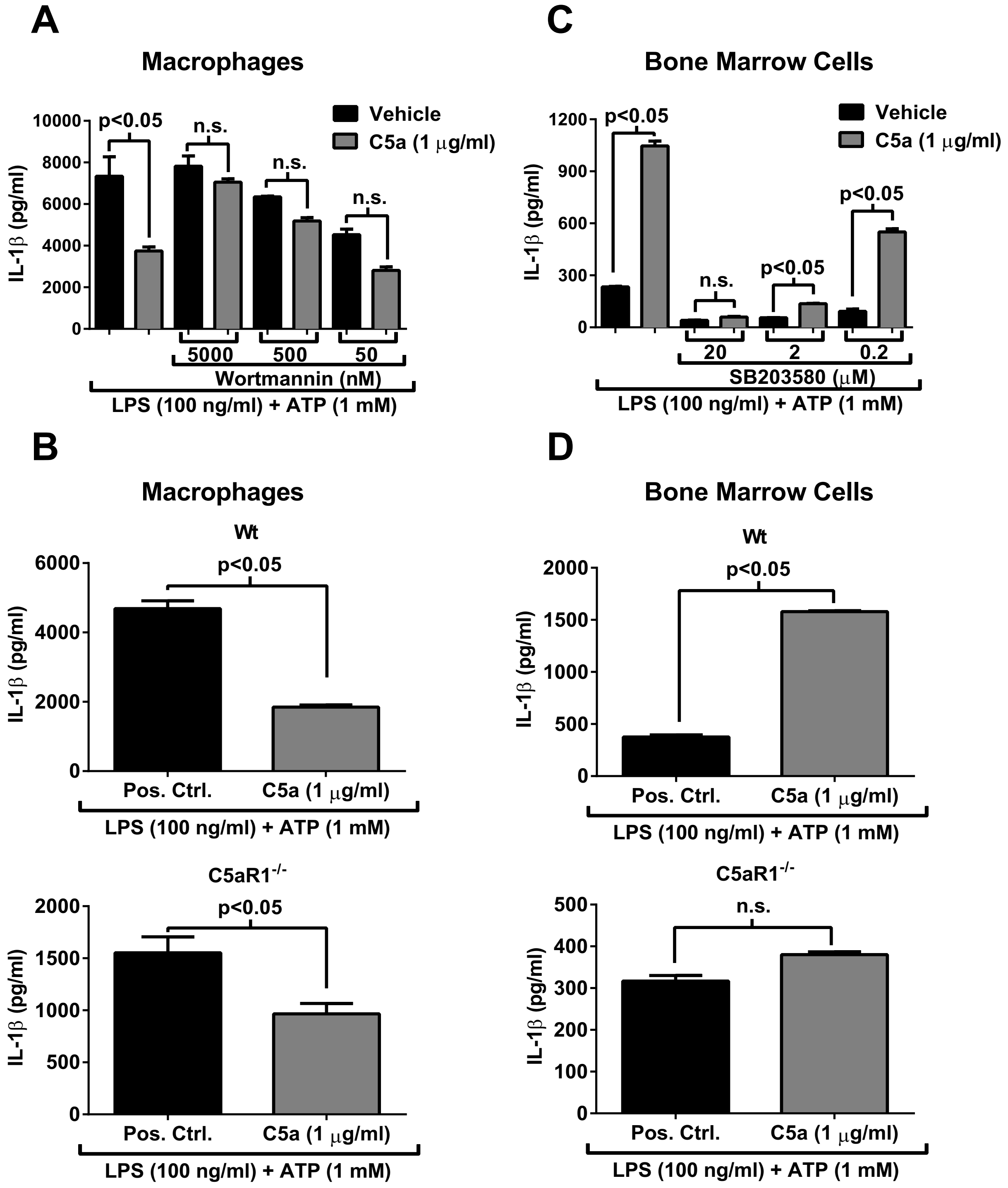

Supp. Fig. 3. IL-1β protein data for C5a signaling and receptor dependency experiments. For signaling inhibition studies (a and c), 1x10^6 peritoneal macrophages (a) or bone marrow cells (c) harvested from wild-type mice were pre-treated with the indicated concentrations of wortmannin (a) or SB 203580 (c) for 1 hour prior to LPS (100 ng/mL) stimulation (4 hours) in the absence or co-presence of C5a (1,000 ng/mL). IL-1β secretion, as quantified by ELISA, was elicited by subsequently treating cells with ATP (1 mM) for 45 minutes. For studies investigating C5aR1 dependency (b and d), 1x10^6 peritoneal macrophages (b) or bone marrow cells (d) harvested either from wild-type or C5aR1$^{-/-}$ mice were treated with LPS (100 ng/mL) in the absence or co-presence of C5a (1,000 ng/mL) for 4 hours. ELISA was used to quantify supernatant IL-1β levels following subsequent ATP (1 mM) treatment for 45 minutes. All protein values are expressed as means +/- standard error of the mean. Experiments were performed in triplicate for ≥ 2 independent experiments, and representative data are shown. Data indicated that C5a requires p38 and C5aR1 for enhancing NLRP3 function in bone marrow cells, but does not fully depend on C5aR1 for suppressing the LPS-primed macrophage IL-1β response, which it regulates via PI3K signaling. Wt = wild type; Pos. Ctrl. = positive control.
